# Will Participatory Syndromic Surveillance Work in Latin America? Piloting a Mobile Approach to Crowdsource Influenza-Like Illness Data in Guatemala

**DOI:** 10.2196/publichealth.8610

**Published:** 2017-11-14

**Authors:** José Tomás Prieto, Jorge H Jara, Juan Pablo Alvis, Luis R Furlan, Christian Travis Murray, Judith Garcia, Pierre-Jean Benghozi, Susan Cornelia Kaydos-Daniels

**Affiliations:** ^1^ Center for Health Studies, Universidad del Valle de Guatemala Guatemala City Guatemala; ^2^ Centre de Recherche en Gestion Centre National de la Recherche Scientifique, Ecole Polytechnique Palaiseau France; ^3^ Center for Studies in Applied Informatics Universidad del Valle de Guatemala Guatemala City Guatemala; ^4^ Ministry of Health Guatemala City Guatemala; ^5^ Influenza Program US Centers for Disease Control and Prevention Guatemala City Guatemala

**Keywords:** crowdsourcing, human flu, influenza, grippe, mHealth, texting, mobile apps, short message service, text message, developing countries

## Abstract

**Background:**

In many Latin American countries, official influenza reports are neither timely nor complete, and surveillance of influenza-like illness (ILI) remains thin in consistency and precision. Public participation with mobile technology may offer new ways of identifying nonmedically attended cases and reduce reporting delays, but no published studies to date have assessed the viability of ILI surveillance with mobile tools in Latin America. We implemented and assessed an ILI-tailored mobile health (mHealth) participatory reporting system.

**Objective:**

The objectives of this study were to evaluate the quality and characteristics of electronically collected data, the user acceptability of the symptom reporting platform, and the costs of running the system and of identifying ILI cases, and to use the collected data to characterize cases of reported ILI.

**Methods:**

We recruited the heads of 189 households comprising 584 persons during randomly selected home visits in Guatemala. From August 2016 to March 2017, participants used text messages or an app to report symptoms of ILI at home, the ages of the ILI cases, if medical attention was sought, and if medicines were bought in pharmacies. We sent weekly reminders to participants and compensated those who sent reports with phone credit. We assessed the simplicity, flexibility, acceptability, stability, timeliness, and data quality of the system.

**Results:**

Nearly half of the participants (47.1%, 89/189) sent one or more reports. We received 468 reports, 83.5% (391/468) via text message and 16.4% (77/468) via app. Nine-tenths of the reports (93.6%, 438/468) were received within 48 hours of the transmission of reminders. Over a quarter of the reports (26.5%, 124/468) indicated that at least someone at home had ILI symptoms. We identified 202 ILI cases and collected age information from almost three-fifths (58.4%, 118/202): 20 were aged between 0 and 5 years, 95 were aged between 6 and 64 years, and three were aged 65 years or older. Medications were purchased from pharmacies, without medical consultation, in 33.1% (41/124) of reported cases. Medical attention was sought in 27.4% (34/124) of reported cases. The cost of identifying an ILI case was US $6.00. We found a positive correlation (Pearson correlation coefficient=.8) between reported ILI and official surveillance data for noninfluenza viruses from weeks 41 (2016) to 13 (2017).

**Conclusions:**

Our system has the potential to serve as a practical complement to respiratory virus surveillance in Guatemala. Its strongest attributes are simplicity, flexibility, and timeliness. The biggest challenge was low enrollment caused by people’s fear of victimization and lack of phone credit. Authorities in Central America could test similar methods to improve the timeliness, and extend the breadth, of disease surveillance. It may allow them to rapidly detect localized or unusual circulation of acute respiratory illness and trigger appropriate public health actions.

## Introduction

### Influenza Surveillance in Latin America

The routine collection, analysis, and reporting of disease and injury data is the foundation of public health: it provides health officials with current information to ground public health decisions and action [1,2]. Despite efforts to improve disease surveillance and response, developing countries still have difficulty in accurately identifying, diagnosing, and reporting infectious diseases [3]. In Latin America, influenza and other respiratory virus surveillance lacks timeliness and is nonspecific, particularly in remote areas [4-7]. The recommendations that were proposed more than a decade ago to advance surveillance [7]—improving the quality of available data, enhancing communication of the information obtained by surveillance activities to stakeholders, and increasing awareness of health authorities of influenza impact in public health—have yet to be fully implemented in the region [5].

Guatemala is illustrative of this challenge. Disease surveillance activities are constrained by scarcity of resources, personnel, and infrastructure [8,9]. Seasonal influenza information is incomplete and delayed because reports are publicly available 2 to 3 weeks after the emergence of medically attended cases from which samples are collected. Low usage of government health facilities conducting sentinel surveillance of influenza makes it difficult to accurately estimate the geographic distribution of influenza in the country [8]. The effect of the data shortcomings is that authorities and providers are forced to make decisions, such as defining the timing of effective vaccination campaigns, using inadequate information on a seasonal disease. Countries in the region facing the same challenges are in need of effective tools to improve the quality and timeliness of surveillance data.

### The Promise of Culturally Relevant Participatory Surveillance

Participatory surveillance strategies, by which individuals report symptoms voluntarily, have the potential to serve as a viable complement to existing outpatient, hospital-based, and laboratory surveillance systems because they can be flexible, sensitive, and accurate [10,11]. Studies suggest that electronic participatory surveillance may mitigate delays in reporting, improve timeliness of surveillance, extend surveillance to unmonitored locations, reduce costs, increase the population size under surveillance, and characterize cases of nonmedically attended illnesses [10,12-19]. Although attention has mainly been focused on using data from Internet posts and search queries for influenza surveillance [18], other approaches to participatory surveillance of influenza, such as Flu Near You [10] (and its extension Salud Boricua [20] in Puerto Rico), InfluenzaNet [21,22], or the Web-based Reporta [23] in Mexico, have generated relevant information by collecting data from users who periodically report symptoms; the data have been used to signal the beginning of yearly influenza epidemics, allowing an appropriate mobilization of communities to seek vaccines and a targeted promotion of nonpharmaceutical interventions such as avoiding close contact or covering the mouth and nose when coughing or sneezing [21].

An electronic bottom-up surveillance system may offer new ways of strengthening situational awareness by engaging citizens in countries such as Guatemala. Implementing functional participatory surveillance systems, however, requires designing systems that are appropriate and scalable, and developing local strategies for encouraging participation [11,24]. In Latin America, the unsuitability of Google Flu Trends to accurately predict influenza outbreaks across countries has been attributed to low Internet penetration rates and to heterogeneous usage of search engines among non-English speaking populations [24]. Alternatively, the ubiquity of feature phones—a midway point between smartphones and basic mobile phones [25], which are only capable of voice calling and text messaging—coupled with the increasing penetration of smartphones provides culturally relevant infrastructure opportunities to capture health information with the use of apps and short message service (SMS) (or text messages) [26,27]; it is almost four times more likely to be a mobile phone user than an Internet user in Central America (34 Internet users per 100 people [28] vs 130 mobile subscriptions [29] per 100 people). No published studies to date have assessed the viability of ILI surveillance with mobile-based tools in the region.

### The Study in Guatemala

We implemented and assessed a participatory, bimodal, mobile phone–based surveillance system for ILI symptoms, the first in the Latin American region. The mobile health (mHealth) system was purposely developed to make use of popular mobile communication tools—SMS and Android apps—in Guatemala. SMS provides a cross-platform mobile communication channel that is widely used in the country, where mobile penetration rates exceed 100% [29]. Android smartphones dominate Central and South American markets with approximately 80% of the market share [30]; in Guatemala, the leading telecom—with 54.5% of active mobile lines—reported that the four most used smartphones in 2016 ran on the Android operating system [28]. We conducted a study between August 2016 and March 2017 in San Marcos, Guatemala, to assess the relevance, performance, and potential of the ILI-tailored reporting system. Specific aims were to evaluate the quality and characteristics of the collected data, the user acceptability of the platform, and the costs of running the system and of identifying ILI cases and to use the collected data to characterize cases of reported ILI in the study population.

## Methods

### The Bimodal Data Capturing System

**Figure 1:**
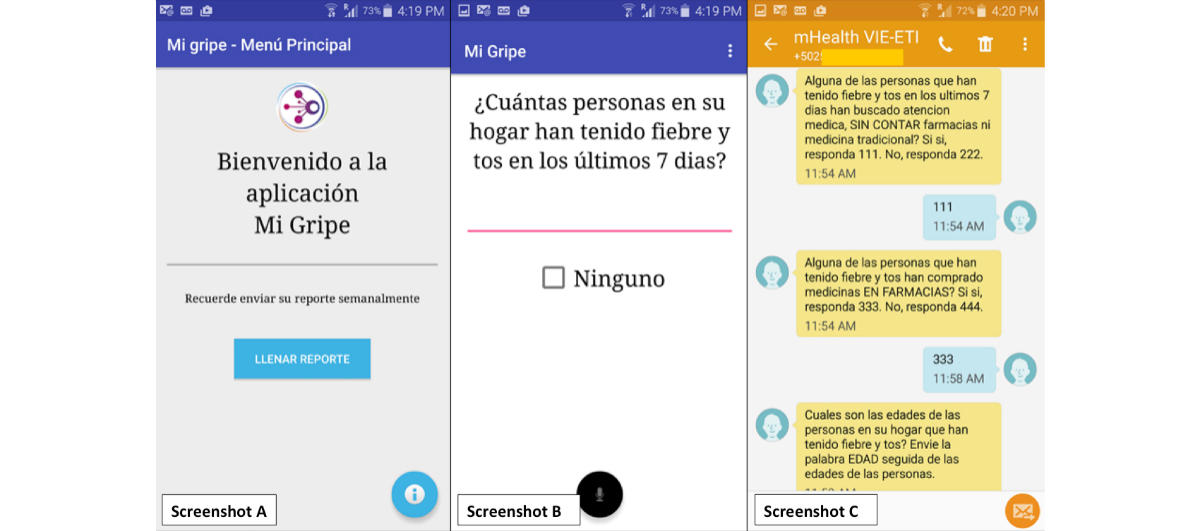
Two screenshots of the app “Mi Gripe” (left and middle) and screenshot of exchanges for short message service (SMS) users (right). Screenshot A: Translation: "Welcome to the Mi Gripe App. Please remember to send your weekly report." Screenshot B: Translation: “How many people at home had fever with cough or sore throat in the last 7 days? None." Screenshot C: Translation: “Did the people who had fever with cough or sore throat in the last 7 days seek medical attention, excluding traditional medicine and pharmacies? Send 111 if yes, 222 if no,” “Did the people who had fever and cough or sore throat in the last 7 days buy medicines at a pharmacy? Send 333 if yes, 444 if no,” “What are the ages of the people who had fever with cough or sore throat? Send AGE followed by their ages.”.

We created a system capable of capturing ILI reports from two sources, SMS and the smartphone app “Mi Gripe” (“My Flu” in Spanish) (see Figure 1 for screenshots). Our ILI case definition was as follows: measured fever and cough or sore throat, with onset within the last 7 days.

We used a Dell Laptop running on Microsoft Windows 7 Professional with an Intel Core Duo processor and 4-GB RAM, a Huawei E173 GSM Modem connected to the laptop, and a subscriber identity module (SIM) card compatible with the GSM Modem. We used the open-source program FrontlineSMS [31] to import contact datasets and to schedule the delivery of weekly reminders. We configured the software to send automatic follow-up questions to participants based on keywords contained in incoming SMSs. The logic for the SMS exchange was as follows: at the beginning of each week, the platform sent the question: “How many people at home had fever with cough or sore throat in the last 7 days? Send 0 if none. Respond with the number if there were any.” If the answer was 0, the system sent a “Thank you” message. If the answer was a number between 1 and 15, the system sent the next question: “Did the people who had fever with cough or sore throat in the last 7 days seek medical attention (excluding traditional medicine and pharmacies)? Send 111 if yes, 222 if no.” If the answer contained either 111 or 222, the system sent the question: “Did the people who had fever and cough or sore throat in the last 7 days buy medicines at a pharmacy? Send 333 if yes, 444 if no.” If the answer contained 333 or 444, the following question was sent automatically: “What are the ages of the people who had fever with cough or sore throat? Send AGE followed by their ages.” If the system identified the keyword AGE in the text, a “Thank you” message was sent, marking the end of a weekly SMS report (see Multimedia Appendices 1 and 2).

We also created the app “Mi Gripe” for Android smartphones. The app “Mi Gripe” allowed participants to complete a weekly report by answering a set of questions (the same ones we used in the SMS modality). A reminder popped up every week to remind users to complete the weekly report. Participants could not skip questions in the app because data entry validation required them to answer questions before moving to the next one. A message was displayed if the report for the week had been sent. Reports were sent if and when the mobile device found connectivity to the Internet.

### Location and Participants

The study was conducted between August 2016 and March 2017 in southwestern Guatemala, close to the Mexican border and the Pacific Ocean coast, in the municipalities of San Marcos and San Pedro (Department of San Marcos).

As part of a health utilization survey conducted in the coverage area of the San Marcos and San Pedro public health centers, we selected 1671 households using a cluster random sample strategy (paper forthcoming). We invited heads of Spanish-speaking households to participate in the study. The inclusion criteria for the study were as follows: households with at least one mobile phone and households where at least one person knew how to read and write in Spanish. Visits were organized as follows: clusters of houses in the coverage area of the health centers were first selected using a probabilistic cluster stratified sampling.

The stratification was based on the location (urban or rural) of houses. Within each cluster, a random sample of households was selected to conduct a health utilization survey, which would help estimate the burden of influenza and other respiratory viruses in sentinel surveillance clinics, with each head of the household. At the end of the 1- to 2-hour survey, the field personnel invited the heads of households to participate in the study.

Interested participants who signed an informed consent form were instructed to send reports once per week via SMS if they had a feature phone or via app if they had a smartphone, using their phone credit. The study’s field personnel installed “Mi Gripe” on smartphones of app participants and saved the reporting number under the contact name “Report symptoms” for SMS participants. Participants were credited with ~US $0.40 for each report sent at the end of each month (to cover the cost of sending 4 SMSs). Maximum compensation was set at ~US $1.60 per month, thus compensating no more than four reports per month.

### Coding of SMS Reports

SMS participants were instructed to follow response rules described at the end of every text message, for example, “Send AGE followed by the ages” (details are provided in Multimedia Appendices 1 and 2). At the design stage, we anticipated that participant reports would not systematically comply with the response rules. We therefore prospectively included variants of possible responses in the automatic response system created in FrontlineSMS to optimize interactions with participants. At the end of the project, investigators manually reviewed all answers and recoded them when necessary to have standardized answers for each question, to calculate response times, and to simplify data aggregation and analysis.

### Analysis

We used descriptive statistics to characterize households and electronic reports and the *t* test to compare means. A significance level of .05 was prespecified for all comparisons. The Pan American Health Organization (PAHO) publishes in the Health Intelligence Platform [32] the weekly number of laboratory-confirmed cases of respiratory viruses. In Guatemala, information is based on samples collected by sentinel surveillance sites: we calculated the Pearson correlation test to investigate the correlation between the number of electronically identified ILI cases in participants’ reports and the official number of reported, laboratory-confirmed cases of influenza and other respiratory viruses (respiratory syncytial virus [RSV], metapneumovirus, adenoviruses, and parainfluenza viruses) published by PAHO, for Guatemala. We hypothesized that there would be a weak to moderate positive correlation (.20≤ *r* ≤.59) between the number of electronically identified ILI cases and the number of reported, laboratory-confirmed cases of respiratory virus during the study period. Costs incurred for text messages, participant compensation, electricity, rental space, and personnel are presented in US dollars. We summarized age information of electronically identified ILI cases by age group of people at high risk for developing flu-related complications (children younger than 5 years and adults 65 years of age and older). Statistical analysis was conducted using STATA, version 13.0 for Macintosh, and Microsoft Excel for Mac 2011. On the basis of the Centers of Disease Control and Prevention (CDC) Updated Guidelines for Evaluating Public Health Surveillance Systems [33], we assessed, in Discussion, the simplicity, flexibility, acceptability, stability, timeliness, representativeness, and data quality of the system. All data generated or analyzed during this study are included in this published paper and its Multimedia Appendices. Further information from the study can be requested from the corresponding author on reasonable request.

### Ethics Approval

This study was conducted in accordance with all applicable local regulatory requirements and the principles of the Declaration of Helsinki. The study was approved by the Ethics Committee of Universidad del Valle de Guatemala on July 5, 2016, and by the Guatemalan National Ethics Committee on August 5, 2016 (resolution 17-2016). Both committees provided ethical oversight during the study.

## Results

### Demographic Information

Approximately one-fifth (20.41%, 341/1671) of visited households were not eligible because the head of household reported not having a mobile phone at home. The majority of eligible households (85.79%, 1141/1330) refused to participate because of security concerns or lack of credit at the time of enrollment. We recruited the heads of 189 eligible households (14.21%, 189/1330), comprising 584 people.

Among participants, 84.1% (159/189) used feature phones, and 15.9% (30/189) used smartphones. During the home visits, 177 households provided demographic information. The mean age of people at participating homes was 28 years and ranged between 0 and 89 years. The mean age of head of households was 40 years (Table 1).

Only 22.0% (39/177) of participants had access to a fixed telephone line at home. Households in the SMS and app modalities were similar: the *t* test revealed no significant differences between SMS and app households in terms of the number of people per home, the ages of people living at home, or the ages of the heads of households.

### SMS and App Reports

From August 2016 to March 2017, we received 468 reports, a mean of 2.5 reports per participant household (Table 2).

**Table 1:** Demographic information of participant households.

Indicator		Modality		Total
		SMS^a^	App	
Number of recruited households		160	29	189
**Number of people per home**		**N=149**	**N=28**	**N=177**
	Mean (IQR^b^)	3 (2-4)	4 (3-5)	3 (2-4)
	Range	1-7	1-6	1-7
**Age of people in household**		**N=149**	**N=28**	**N=177**
	Mean	29	27	28
	Median (IQR)	26 (15-42)	23 (15-40)	25 (25-42)
	Minimum-maximum	0-89	0-73	0-89
**Age of head of household**		**N=151**	**N=26**	**N=177**
	Mean	40	40	40.3
	Median (IQR)	39 (28-51)	39 (25-50)	39 (28-50)
	Minimum-maximum	18-83	20-68	18-83
**Percentage of homes with**		**N=151**	**N=26**	**N=177**
	Electricity, n (%)	148 (98.0)	26 (100)	98.3 (17.4)
	Running water, n (%)	150 (99.3)	25 (96)	175 (98.9)
	Fixed telephone line, n (%)	33 (21.8)	6 (23)	39 (22.0)
**Education of head of household**		**N=141**	**N=25**	**N=166**
	No studies, n (%)	4 (2.8)	1 (4)	5 (3.0)
	Some primary school, n (%)	42 (30.0)	5 (20)	47 (28.3)
	Some secondary school, n (%)	26 (18.4)	4 (16)	30 (18.1)
	Some university, n (%)	69 (48.9)	15 (60)	84 (50.6)

^a^SMS: short message service.

^b^IQR: interquartile range.

**Table 2:** Reporting from August 2016 to March 2017.

Indicator		SMS^a^	App	Total
**Reports per user**				
	Mean	2.4	2.6	2.5
	Minimum-maximum	0-25	0-25	0-25
**Participants who**				
	Sent no reports, n (%)	85 (53.1)	15 (51.7)	100 (52.9)
	Sent between 1 and 5 reports, n (%)	56 (35.0)	11 (37.9)	67 (35.5)
	Sent between 6 and 15 reports, n (%)	9 (5.6)	1 (3.5)	10 (5.3)
	Sent 16 or more reports, n (%)	10 (6.3)	2 (6.9)	12 (6.3)
**Age group of ILI^b^cases**				
	0-5 years	13	7	20
	6-64 years	73	22	95
	>64 years	0	3	3

^a^SMS: short service message.

^b^ILI: influenza-like illness.

More than four-fifths (83.5%, 391/468) of reports were received via SMS and the remainder (16.4%, 77/468) via the app “Mi Gripe.” Almost nine-tenths (88.0%, 412/468) of reports were received within 24 hours and 93.6% (438/468) within 48 hours of transmission of weekly reminders. Nearly half (47.1%, 89/189) of participants sent one or more reports. Moreover, 12 participants (6.3%, 12/189) sent 16 or more reports during the study.

**Figure 2:**
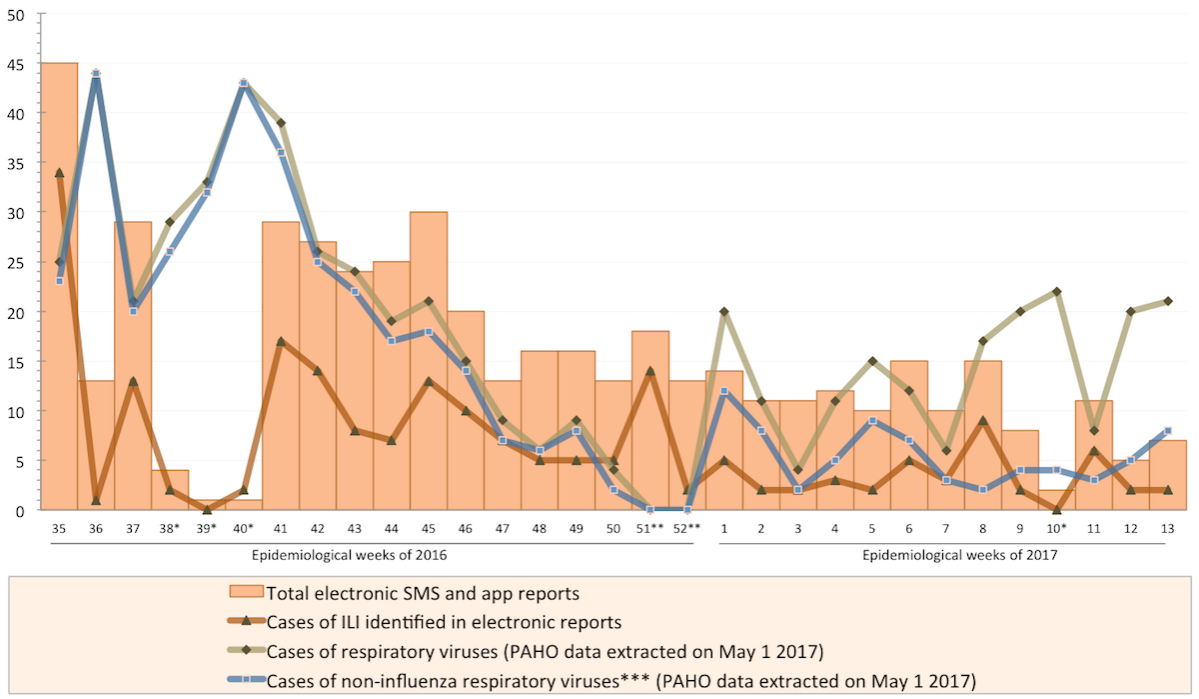
Electronically-identified influenza-like illness (ILI) in San Marcos and official respiratory virus surveillance data in Guatemala, 2016 and 2017. *Weekly reminders were not sent to participants from weeks 38 to 40, when the project was interrupted. In week 10 of 2017, the GSM modem was accidentally disconnected from the short message service (SMS) platform and the scheduled reminders were not sent. **Official respiratory virus surveillance activities are normally stopped the last 2 weeks of every year. ***Noninfluenza respiratory viruses for the considered period include the following: respiratory syncytial virus, metapneumovirus, adenovirus, and parainfluenza virus.

Nearly three quarters of reports (73.5%, 344/468) indicated that no one at home developed ILI symptoms during the project period. Over a quarter of reports indicated that someone at home developed ILI symptoms (26.5%, 124/468). The 124 reports indicated that 202 individuals presented ILI symptoms during the study (see Figure 2 for weekly details about the number of reported ILI episodes to the electronic platform and the number of official laboratory-confirmed cases of respiratory viruses in Guatemala).

We received age data for 118 of those 202 cases: the majority (80.5%, 95/118) were aged between 6 and 64 years, 16.9% (20/118) were aged between 0 and 5 years, and three (2.6%, 3/118) were 75 years old. Most ILI cases for which we had age data (89.0%, 105/118) occurred among family members rather than the respondent: in 105 cases, the ages of the ILI cases did not correspond to the ages of the heads of households.

According to PAHO data [32], the number of reported, laboratory-confirmed cases of influenza in Guatemala started to increase in epidemiological weeks 6 and 7 of 2016 and started to decrease in week 12, with the end of the influenza season around week 17 of 2016. The number of reported, laboratory-confirmed cases of RSV, metapneumovirus, adenovirus, and parainfluenza started to increase between epidemiological weeks 20 and 23 of 2016 and started to decrease between weeks 40 to 41 of 2016, with the end of the season around week 1 of 2017.

We found a positive correlation (Pearson correlation coefficient=.8) between the number of ILI cases reported to our system and the number of reported, laboratory-confirmed cases of noninfluenza respiratory viruses (RSV, metapneumovirus, adenovirus, and parainfluenza virus) in Guatemala, published by PAHO from epidemiological week 41 in 2016 to 13 in 2017. The correlation was weaker (Pearson correlation coefficient=.6) when we combined the number of reported, laboratory-confirmed cases of influenza and other respiratory virus cases (Table 3).

We found a negative correlation (*r*=−.3) between the number of ILI cases reported to our system and the number of reported, laboratory-confirmed cases of influenza published by PAHO. No correlation was found between positivity for electronically identified ILI (defined as the number of electronically identified cases of ILI divided by the total number of household members involved in weekly reports) and positivity for laboratory-confirmed respiratory viruses in Guatemala in 2016 and 2017 (Multimedia Appendix 3).

**Table 3:** Pearson correlation between electronically identified influenza-like illness (ILI) in San Marcos and San Pedro and respiratory virus laboratory data published by the Pan American Health Organization for Guatemala.

Variables	Pearson correlation, *r*	
	From epidemiological week 41 in 2016 to 13 in 2017^a^	From epidemiological week 41 in 2016 to 13 in 2017 (excluding 10, 51, and 52)^b^
Influenza and electronically identified ILI^c^	−.412	−.342
Noninfluenza respiratory viruses and electronically identified ILI	.656	.802
Influenza and other respiratory viruses and electronically identified ILI	.373	.623

^a^The project started in epidemiological week 35 of 2016 and was temporarily interrupted from epidemiological weeks 38 to 40. We considered data from week 41 in 2016 to 13 in 2017 to conduct the correlation analysis.

^b^We excluded weeks 51 and 52 of 2016 because official respiratory virus surveillance activities are stopped during the last 2 weeks of the year, and no official surveillance data are published. We excluded week 10 of 2017 because the GSM modem was accidentally disconnected from the SMS platform and the scheduled reminders were not sent.

^c^ILI: influenza-like illness.

### Costs

Running the ILI surveillance system for 7 months cost approximately US $1212.58: one mHealth operator monitored the electronic platforms 3 hours per week during the study, amounting to US $738.21 in personnel; the system sent 5147 text messages, amounting to US $275.24; we reimbursed participants who sent a complete report either via SMS or the app, amounting to US $158.02; and the laptop and the server receiving reports were turned on 24 hours a day for 212 days, amounting to US $5.77 in electricity and US $35.33 in rental space. The cost of identifying one new ILI case in electronic reports was US $6 (details are provided in Multimedia Appendix 4).

## Discussion

### Summary

The results show the ability of the developed system to capture ILI data that could be used to improve the timeliness and expand the breadth of syndromic surveillance activities. The data collected could be used to rapidly detect localized or unusual circulation of acute respiratory illness. The strategy could be further explored to determine the start of epidemic activity of respiratory viruses each year. This would allow authorities to trigger appropriate public health action such as collecting samples outside of normal surveillance, increasing communication to the public about the need to vaccinate target groups, and promoting nonpharmaceutical interventions to prevent transmission of viruses. The pilot allowed us to monitor the performance of the participatory surveillance system and assess some of its attributes, particularly its simplicity, flexibility, acceptability, stability, timeliness, representativeness, and data quality.

### Simplicity, Flexibility, and Timeliness

The system was designed to maximize ease of operation. By using autonomous processes (SMS and app-driven), we minimized time spent on maintaining and monitoring the system. The reporting platform was sufficiently stable: it remained fully functional for 7 months, except for 1 week in March 2017, when the GSM modem was accidentally disconnected from the SMS platform. The reporting platform was accessible through the most popular mobile communication methods in the region. From the user’s perspective, it was designed to be intuitive to maximize acceptability. Nearly half of the participants (47.1%, 89/189) sent at least one report. In total, we received 468 reports through SMS and the app. The rate of long-term, consistently reporting participants in our project (those who sent 16 reports or more) was lower than in a similar project in the United States [10] (6.3% vs 10.4%).

An important characteristic of the system was its timeliness: it generated data more rapidly than official surveillance methods. Almost all (93.6%, 438/468) ILI reports were received within 48 hours of transmission of weekly reminders, whereas the average time between symptoms onset and laboratory tests in sentinel surveillance sites in Guatemala was 161 hours (almost 7 days) in 2014 (data provided by the Department of Epidemiology of the Guatemalan Ministry of Health). The PAHO surveillance data that we used to calculate correlations took 2 to 3 weeks to become public during the study period. In a scaled-up implementation, this approach would allow the enactment of immediate actions while waiting for laboratory results.

Results were derived from reports sent by the heads of participant households. The coding of SMS reports revealed that, even when instructed to do so, the reporters did not consistently comply with the predefined response rules. During the study, we prospectively adjusted keyword-based responses to gradually improve interactions with participants and increase the flexibility of the system. At the end of the project, we recoded the remaining uncategorized reports. Due to the informational benefits of each SMS—rule-compliant or not—associated efforts in developing tools that assist in the interpretation of their meaning are justified. A useful participatory electronic system must be able to capture structured and unstructured data, as well as expected and unexpected data, and transform them into relevant high-quality information in a timely manner [34]. A future version of the system could use this knowledge to train machine-learning algorithms that would automatically code incoming reports and self-increase flexibility. This would enable the generation of real-time data graphs and statistics for epidemiologists and public health experts. With modest development and configuration efforts, the approach may also make it possible to monitor other syndromes and contribute to the identification of febrile disease outbreaks in the region, such as dengue, chikungunya, or zika. It may also contribute to improved response to disease outbreaks by providing near real-time syndromic data and rapidly detecting localized and unusual incidence of specific symptoms [35-37]. Such information could prompt public health authorities to focus resources on actively sampling cases to determine the etiology of an ILI cluster.

### Representativeness, Data Quality, and New Information

The pilot surveillance system was not designed to be representative; it was a proof-of-concept for participatory surveillance in Guatemala. Reports submitted during the study may in fact not be representative of all Guatemalan households either in terms of the incidence of ILI or of willingness to participate in the participatory system. One-seventh (14.21%, 189/1330) of eligible households accepted to participate in the study. The low uptake is consistent with that of similar projects [10] but might be explained by three different factors. First, participants were invited to the project after answering a 1- to 2-hour-long survey. Individuals were fatigued by the lengthy questionnaire and only occasionally wanted to hear about the project. Second, the area of San Marcos in Guatemala is particularly affected by gang violence. The heads of households often communicated their concerns about security to the field personnel when deciding to participate. Third, participants were told that they would be compensated for every report they would send during the study; however, many of them did not have phone credit at enrollment and were not interested in being reimbursed at the end of the month. A more effective strategy could have been to invite participants to the study before conducting the survey, give credit to interested participants before sending reminders, and let participants handle the installation of the app or the saving of the reporting number.

Reports suggest that on 41 occasions (33.1% of reported ILI cases; 41/124), people at home self-medicated and only 27.4% (34/124) of participants who reported ILI symptoms sought medical attention (vs 35% for Flu Near You users [38]). On 50 occasions (40.3% of reported ILI cases; 50/124), medical attention was not sought, thus impeding the collection of samples and a laboratory confirmation of their diagnosis. The project generated information during epidemiological weeks 51 and 52 of 2016, when no official data were published for any respiratory virus in Guatemala [32]. This new information about health-seeking behavior during ILI episodes may be used to estimate the burden of the syndrome in the San Marcos department using recent modeling methods [39,40]; with additional representative data coming from more locations, it might also be used to estimate the burden of ILI in the country, which is likely substantial [41,42]. The approach presented here could contribute to strengthen disease surveillance activities in neighboring countries (Honduras, El Salvador, Nicaragua, and Belize) that have similar challenges in conducting influenza and other respiratory virus surveillance. As highlighted in a recent scientific commentary [43], crowdsourcing could make governments more agile in responding to public health problems. The strategy echoes the European Influenzanet in its ability to monitor ILI in the population, including individuals who do not seek medical attention [22].

Retrospective credit compensation and weekly reminders were used to secure the periodic contributions of participants. The importance of weekly reminders was serendipitously demonstrated; from weeks 39 to 41 in 2016 and in week 10 in 2017, reminders were not sent to participants and the number of reports decreased dramatically (Figure 2). But even when weekly reminders were sent, results reveal that more than half (52.9%, 100/189) of participants did not send reports and almost one quarter (23.3%, 44/189) sent one or two reports only during the study. The finding suggests that new sustainable arrangements and incentives should be tested to motivate a continued participation. The feeding of weekly professional health information in exchange for reports and gamification methods, for example, could be effective ways of engaging citizens in the participatory system [44-46], especially in Latin American countries where access to public health centers is challenging and the demand for health information is expected to intensify [47]. Future systems should exploit lessons learned from related systems, such as Flutracking in Australia [48], to engage and motivate sustained participation. Some of these lessons seem relevant for the Latin-American context (eg, collect minimum answers and invite participants to report on household members [48]), but others (eg, use social media and website analytics or leverage organizational email invitations [48]) should be further explored to create a culturally appropriate and sustainable system in the region.

### Electronic Participatory ILI Surveillance Opportunities in the Region

The collected data allowed us to identify 202 cases of ILI at home at a cost of less than US $6.00 per identified case. We did not find a strong correlation between the number of electronically identified ILI and the number of reported, laboratory-confirmed cases of influenza, probably because the 2016 flu season ended around May in Guatemala [32]; however, we found a strong positive correlation (*r*=.8) between the number of electronically identified ILI and the number of reported, laboratory-confirmed cases of noninfluenza respiratory viruses cases from epidemiological week 41 in 2016 to 13 in 2017 (Table 3; Figure 2), precisely during a period of increased circulation of noninfluenza respiratory viruses. The study shows the viability of capturing valuable citizen-sourced health data and seems more culturally appropriate than other recently studied alternatives [24,30] in the Latin-American realm. Our choice of reporting platforms, SMS and an Android app, maximizes the chances of having a culturally appropriate solution and minimizes additional software development efforts in a scale-up scenario for Central America. We plan to apply lessons learned and measure associations between self-reported ILI and laboratory-confirmed respiratory virus surveillance in a forthcoming, larger-scale crowdsourcing project.

## Appendix

Multimedia Appendix 1 Processes for the app reporting platform.

Multimedia Appendix 2 Processes for the SMS reporting platform.

Multimedia Appendix 3 Positivity for electronically identified ILI (defined as the number of electronically identified cases of ILI divided by the total number of household members involved in weekly reports) and positivity for laboratory-confirmed respiratory viruses in Guatemala 2016 and 2017.

Multimedia Appendix 4 Dataset and main calculations.

## Abbreviations

CDC: Centers of Disease Control and Prevention
ILI: influenza-like illness
IQR: interquartile range
mHealth: mobile health
PAHO: Pan American Health Organization
RSV: respiratory syncytial virus
SIM: subscriber identity module
SMS: short message service
UVG: Universidad del Valle de Guatemala
