# Supplementary figures and images for: Will Participatory Syndromic Surveillance Work in Latin America? Piloting a Mobile Approach to Crowdsource Influenza-Like Illness Data in Guatemala
Source: JMIR Public Health Surveill. 2017 Nov 14;3(4):e87. doi: 10.2196/publichealth.8610 (PMC5705859; doi:10.2196/publichealth.8610)

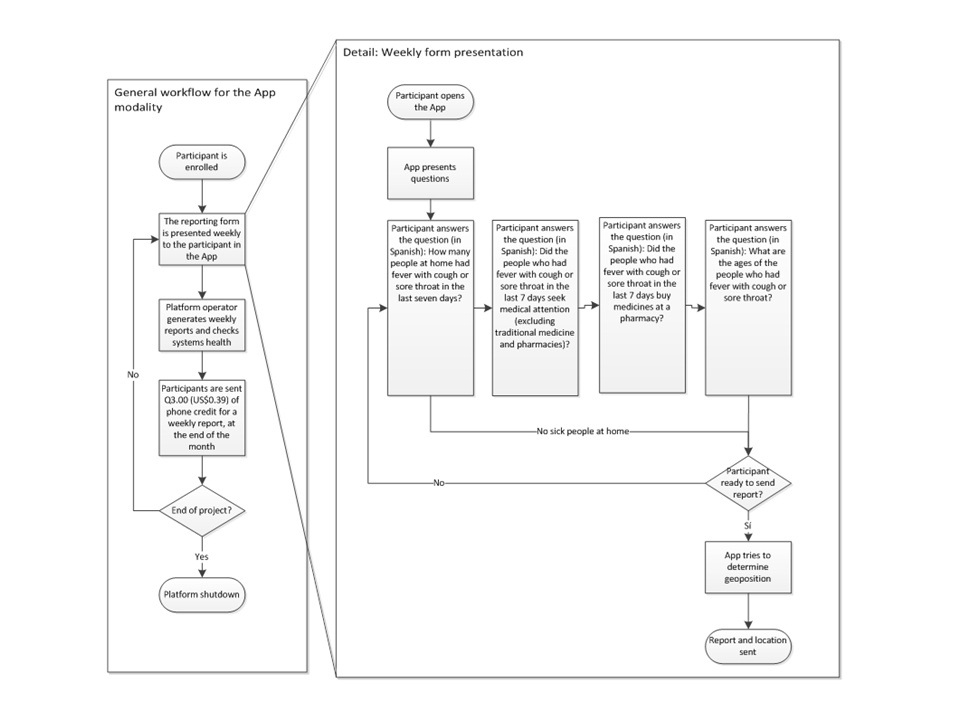

Supplement: Multimedia Appendix 1 [file publichealth_v3i4e87_app1.png]

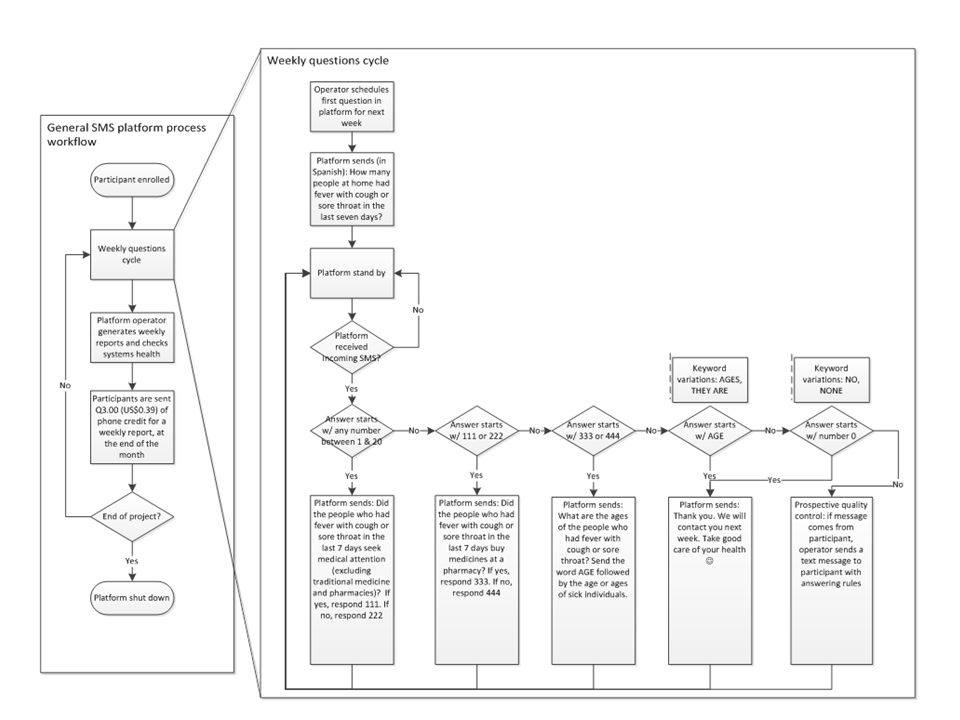

Supplement: Multimedia Appendix 2 [file publichealth_v3i4e87_app2.png]

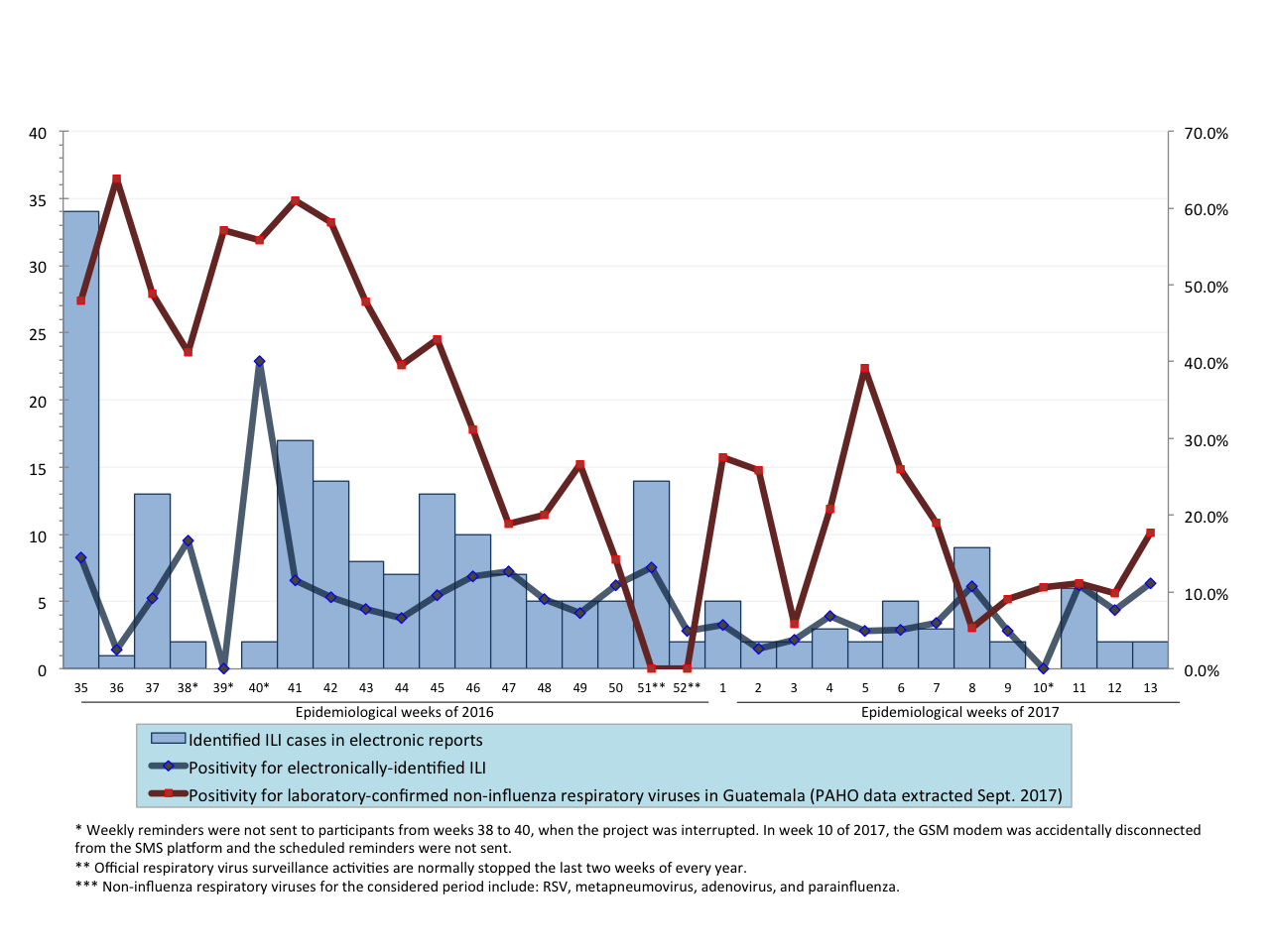

Supplement: Multimedia Appendix 3 [file publichealth_v3i4e87_app3.png]
